# Rapid forest clearing in a Myanmar proposed national park threatens two newly discovered species of geckos (Gekkonidae: *Cyrtodactylus*)

**DOI:** 10.1371/journal.pone.0174432

**Published:** 2017-04-12

**Authors:** Grant M. Connette, Patrick Oswald, Myint Kyaw Thura, Katherine J. LaJeunesse Connette, Mark E. Grindley, Melissa Songer, George R. Zug, Daniel G. Mulcahy

**Affiliations:** 1 Conservation Ecology Center, Smithsonian Conservation Biology Institute, Smithsonian Institution, Front Royal, Virginia, United States of America; 2 Fauna & Flora International, San Chaung Township, Yangon, Myanmar; 3 Myanmar Environment & Sustainable Conservation Co., LTD (MESC), Yangon, Myanmar; 4 Department of Vertebrate Zoology, National Museum of Natural History (NMNH), Smithsonian Institution, Washington D.C., United States of America; 5 Global Genome Initiative (GGI), National Museum of Natural History (NMNH), Smithsonian Institution, Washington D.C., United States of America; Chinese Academy of Forestry, CHINA

## Abstract

Myanmar’s recent transition from military rule towards a more democratic government has largely ended decades of political and economic isolation. Although Myanmar remains heavily forested, increased development in recent years has been accompanied by exceptionally high rates of forest loss. In this study, we document the rapid progression of deforestation in and around the proposed Lenya National Park, which includes some of the largest remaining areas of lowland evergreen rainforest in mainland Southeast Asia. The globally unique forests in this area are rich in biodiversity and remain a critical stronghold for many threatened and endangered species, including large charismatic fauna such as tiger and Asian elephant. We also conducted a rapid assessment survey of the herpetofauna of the proposed national park, which resulted in the discovery of two new species of bent-toed geckos, genus *Cyrtodactylus*. We describe these new species, *C*. *lenya* **sp. nov**. and *C*. *payarhtanensis* **sp. nov**., which were found in association with karst (i.e., limestone) rock formations within mature lowland wet evergreen forest. The two species were discovered less than 35 km apart and are each known from only a single locality. Because of the isolated nature of the karst formations in the proposed Lenya National Park, these geckos likely have geographical ranges restricted to the proposed protected area and are threatened by approaching deforestation. Although lowland evergreen rainforest has vanished from most of continental Southeast Asia, Myanmar can still take decisive action to preserve one of the most biodiverse places on Earth.

## Introduction

Habitat loss due to human land use is a primary driver of species extinctions worldwide [1, 2]. In spite of high rates of new species discoveries in recent years [3, 4], undocumented extinctions of species which were never formally described likely represent a substantial “hidden” component of ongoing global biodiversity loss [5]. Other species are believed to have gone extinct shortly after their initial discovery [6, 7]. Concern over biodiversity loss has therefore led to intensified efforts to describe as many species as possible before it is too late for targeted conservation action [8, 9]. In addition to improved genetic techniques for distinguishing between morphologically-similar “cryptic” species, a key driver of new species discoveries has been increased access to species with limited geographic ranges in previously remote or inaccessible areas [3, 4]. However, species occupying very small ranges may be especially difficult to discover and extremely vulnerable to habitat loss, making them more likely to be threatened by extinction than more widespread species [10–12].

Tropical forests of Southeast Asia are home to an incredible diversity of species and high rates of local endemism [13, 14], but are also experiencing deforestation rates higher than in any other major tropical area [15, 16]. Once cleared, these forests are most commonly replaced by agricultural or agroforestry crops [17, 18] that sustain less biodiversity than natural forests [19, 20]. Lowland forests, which support unique plant and animal communities, have experienced especially high rates of forest loss due to their greater accessibility and proximity to areas of higher human population density [21, 22]. Myanmar is part of the Indo-Burma biodiversity hotspot [13] and retains one of the largest forest areas in Southeast Asia [23], including large tracts of biologically-rich lowland wet evergreen forest [24, 25]. These lowland forests are still home to a number of globally-threatened species such as tiger (*Panthera tigris*), Asian elephant (*Elephas maximus*), Malayan tapir (*Tapiris indicus*), and the world’s last viable populations of Gurney’s Pitta (*Pitta gurneyi*) [26, 27].

Myanmar has an ambitious policy target of including 10% of the country’s area in its Protected Area System by 2030, with the overarching goals of preserving biodiversity and unique ecosystem types [28]. However, lowland wet evergreen forest is currently underrepresented in the Protected Area System [28], and long delays in formally designating protected areas have corresponded with an ongoing period of intense deforestation countrywide. Myanmar had the third highest extent of forest loss by area globally from 2010–2015 [23], and recent annual rates of forest loss for primarily closed-canopy ‘intact forest’ are much higher [25]. A critical area for securing the future of Myanmar’s lowland wet evergreen forest is the proposed Lenya National Park in Tanintharyi Region [29]. A 1766 km^2^ area was first proposed for protected area status in 2002, while the Lenya National Park Extension was proposed in 2004 and would add an additional area of 1399 km^2^ [28]. These two areas contain extensive mature lowland forest as well as large monolithic karst formations, which harbor distinct assemblages of limestone-adapted plants and are themselves islands of unique biodiversity throughout Southeast Asia [30]. Non-state armed groups still contest the national government’s authority in much of Tanintharyi, including the proposed Lenya National Park and Extension area, although recent ceasefire agreements have allowed for increased socioeconomic development in the area [29, 31]. This governance situation currently complicates conservation efforts while both large-scale commercial agriculture and expanding village agroforestry areas are responsible for recent forest loss in the region, including within the boundaries of the proposed Lenya National Park.

In this study, we conducted comprehensive mapping of deforestation in and around the proposed Lenya National Park with the goal of assessing the extent of recent habitat loss and fragmentation faced by the region’s threatened wildlife. We also describe two new species of *Cyrtodactylus* geckos discovered during recent herpetofauna surveys in the proposed Lenya National Park and Lenya National Park Extension. These new species discoveries are further confirmation of the highly biodiverse, and poorly inventoried, nature of southern Myanmar’s forests. The genus *Cyrtodactylus* is a species-rich group of tropical gekkonid lizards that currently includes greater than 200 described species [32], many of which were recently discovered in Southeast Asia (e.g., [33, 34, 35]). These species, and closely related *Cnemaspis* species, are often restricted to isolated karst formations or have limited geographic distributions, which increases their vulnerability to local habitat loss or alteration [36]. Many new *Cyrtodactylus* are also strikingly patterned and may be vulnerable to over-exploitation for the pet trade [37]. The two new species described in this study were found near areas of recent forest clearing, highlighting the potential risk already facing the unique biodiversity in this region due to habitat loss and fragmentation.

## Materials and methods

### Deforestation mapping

We performed visual inspection of freely-available Landsat satellite imagery to identify areas of recent forest loss in and around the proposed Lenya National Park and Lenya National Park Extension. Our focal area included the entire extents of the existing Lenya Reserve Forest, Nga Wun Reserve Forest, and Nga Wun Reserve Forest Extension. Collectively, these government forest reserves encompass the total area originally reported for the proposed Lenya National Park and Lenya National Park Extension [28]. We also examined landscape change in the broader landscape by extending our assessment of deforestation to include a 10 km buffer surrounding these proposed protected areas in Myanmar.

We performed visual interpretation of Landsat imagery and manual digitizing of deforested areas based on a combination of characteristics such as color, texture, patch shape, and patch size. In comparison with model-based classifications relying on spectral information (e.g., [16, 29]), our manual process allowed us to separate areas of agroforestry plantation from forest with high confidence for our limited area of interest around the proposed Lenya National Park. We manually digitized areas where forest was cleared during four separate time periods: 1) prior to 2002; 2) 2002–2009; 3) 2010–2013; and 4) 2014–2016. Areas of non-forest prior to 2001 were digitized based on pan-sharpened Landsat 7 ETM+ imagery from December, 2000. Deforestation from 2001–2009 was identified using Landsat 5 TM imagery from December, 2009. Deforestation occurring from 2010–2013 and 2014–2016 was identified using pan-sharpened Landsat 8 OLI imagery from December, 2013 and May, 2016. Because of the relatively short time period evaluated (2002–2016), we counted any patch cleared as forest loss regardless of whether initial stages of forest regeneration were subsequently allowed to occur. Thus, areas identified as deforested would include shifting cultivation (i.e., slash-and-burn) and logged but unplanted areas of agroforestry plantations because it remains unclear whether these areas will be allowed to revert back to natural forest.

### Herpetofauna sampling methods

**Fig 1:**
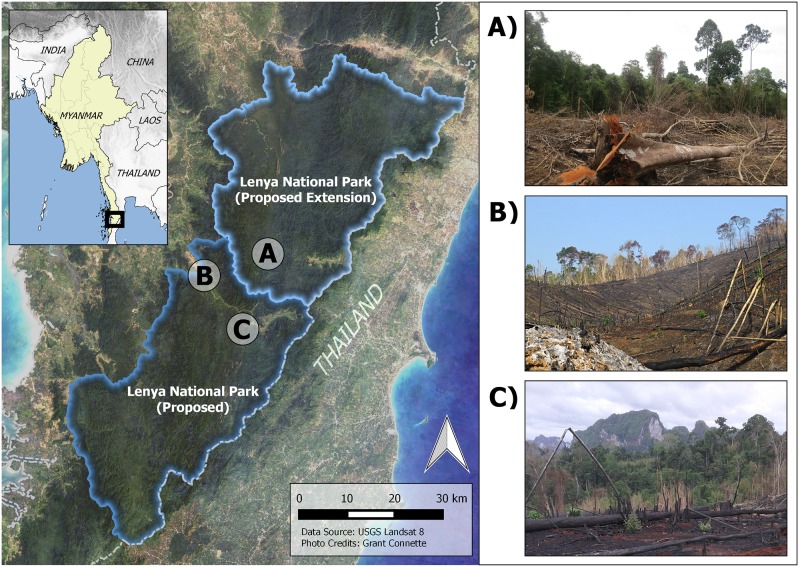
Map of the study landscape (left) showing the proposed Lenya National Park (dark blue) and the proposed Lenya National Park Extension (light blue). Panels A–C show recently deforested areas within the proposed park boundaries.

We conducted two rapid assessment herpetofauna surveys of the proposed Lenya National Park and Lenya National Park Extension in 2015 and 2016. *Cyrtodactylus* species are targeted by the pet-trade and thus vulnerable to over-collecting because of their small geographic distributions, in addition to threats caused by habitat loss. Therefore, we refrain from disclosing their precise localities, but will make this information available to fellow scientists and the necessary government agencies, following procedures for other recently described similar species [38]. We provide purposefully vague latitude and longitude coordinates as centroids of the proposed Lenya National Park and Lenya National Park Extension in the species accounts below and in GenBank and BOLD. The 2015 survey was conducted between 13 May 2015 and 30 May 2015 at two locations in the proposed Lenya National Park (Fig 1). Surveys in 2016 were conducted between 05 May 2016 and 24 May 2016 at one location in the proposed Lenya National Park Extension and two locations in the proposed Lenya National Park (Fig 1). At each location, we searched for reptiles and amphibians along small roads, trails, streams, and karst outcrops at multiple sites within 10 km of our base camp. Much of the forest in the area was selectively logged around 20 years previously but typically was characterized by a mature (> 70 years) broadleaf evergreen overstory with intermittent bamboo stands (*Bamboosa burmanica*).

### Ethics statement

Fieldwork (including non-private land access) was conducted under a Memorandum of Understanding between Myanmar’s Ministry of Natural Resources and Environmental Conservation and Fauna & Flora International (FFI); permitted by Myanmar Forest Department Letter No. 2732. All sampling and collection procedures were reviewed as part of the process of obtaining a field permit. The collection of vertebrates was also reviewed and approved by the Smithsonian Institution, Natural History Building-Animal Care and Use Committee (NHB-ACUC); approval form 2014–02; valid through 2017. Specimens were hand-collected and euthanized with 20% benzocaine. Liver and muscle tissue samples were collected in the field during specimen preparation and preserved separately in a DMSO/EDTA salt-saturated buffer [39]. Specimens were subsequently fixed in 10% formalin and transferred to 70% ethanol for long-term storage at the Smithsonian Institution's United States National Museum (USNM) collection, housed at the National Museum of Natural History (NMNH), and at the California Academy of Sciences (CAS). All specimens may be accessed by other researchers at USNM (specimen numbers: 587408–587411, 587788–587789, 587791–587792) and CAS (specimen numbers: 260232–260233).

### Nomenclatural acts

The electronic edition of this article conforms to the requirements of the amended International Code of Zoological Nomenclature, and hence the new names contained herein are available under that Code from the electronic edition of this article. This published work and the nomenclatural acts it contains have been registered in ZooBank, the online registration system for the ICZN. The ZooBank LSIDs (Life Science Identifiers) can be resolved and the associated information viewed through any standard web browser by appending the LSID to the prefix "http://zoobank.org/". The LSID for this publication is: urn:lsid:zoobank.org:pub:C8862B92-82E7-495F-88FE-60A01C21D1F3. The electronic edition of this work was published in a journal with an ISSN, and has been archived and is available from the following digital repositories: PubMed Central, LOCKSS.

### Molecular analyses

Extractions of genomic DNA were conducted on an Auto-Genprep 965 (2011 AutoGen, Inc.), using standard phenol manufacturer protocols. Genomic DNA was eluted in 100 μl of AutoGen R9 re-suspension buffer. We sequenced the DNA barcode 5' region of the COI mtDNA locus using the ReptBCf-r primers [40] and Chmf4-r4 [41] in 10 μl using the protocol in Table 2 of [42]. Because there are a large number of ND2 mtDNA sequences available in GenBank, we include the ND2 locus using the primers metF6–COIR1 [43]. Cycle-sequence reactions were performed in both directions, using the PCR primers, and an internal reverse primer LVT5617 [44] for ND2 using BigDye Terminator v3.1 Cycle Sequencing Kit's in 0.25 x 10 μl reactions run on and ABI3730 Sequencer (2011 Life Technologies). Raw trace files were edited in Geneious 9.1.5 (Biomatters Ltd 2005–2016), complementary strands were aligned, edited, and inspected for translation. Consensus sequences were aligned with samples from GenBank for the respective loci in Geneious using the MUSCLE Alignment with default settings and secondarily inspected for codon alignment and translation. The ND2 locus required some manual adjustments to insure codon translation and the associated tRNA region was omitted because it was lacking or incomplete for many taxa in GenBank. Maximum-likelihood analyses were conducted on each gene separately in RAxML v8.2 [45] using the rapid-bootstrap (100 replicates) plus best likelihood tree in a single search option, under the GTR nucleotide substitution model with each gene as a single partition. Trees were rooted at midpoint post-analyses for graphical representation. We generated COI and ND2 trees to simply compare our sequences to those in GenBank, aware that these short mtDNA reads are likely inadequate to fully resolve a *Cyrtodactylus* evolutionary history. Sequences generated for this study were deposited in GenBank under the accession numbers KY041652–KY041668, and COI sequences included original trace files and metadata in order to receive the keyword "barcode" in GenBank and were also submitted to the Barcode of Life Database (BOLD: MYARC001-16 to MYARC010-16).

## Results

### Extent of deforestation

**Fig 2:**
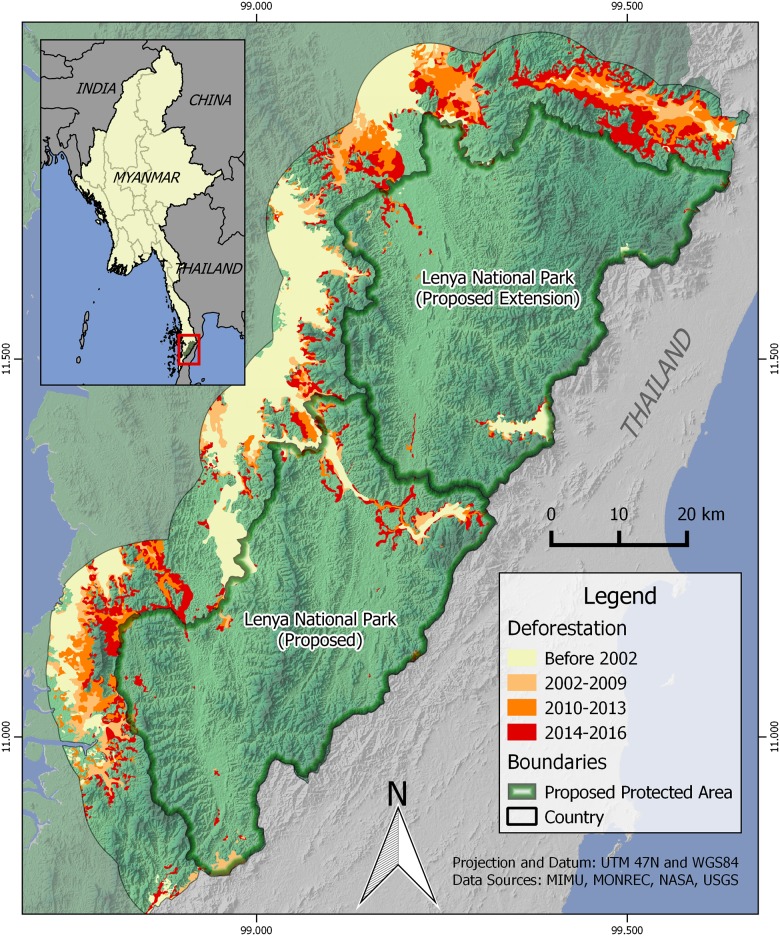
Deforestation in and around the proposed Lenya National Park (currently the Lenya and Nga Wun Reserve Forests). The highlighted focal region includes the proposed protected area as well as surrounding areas in Myanmar within 10 km.

**Fig 3:**
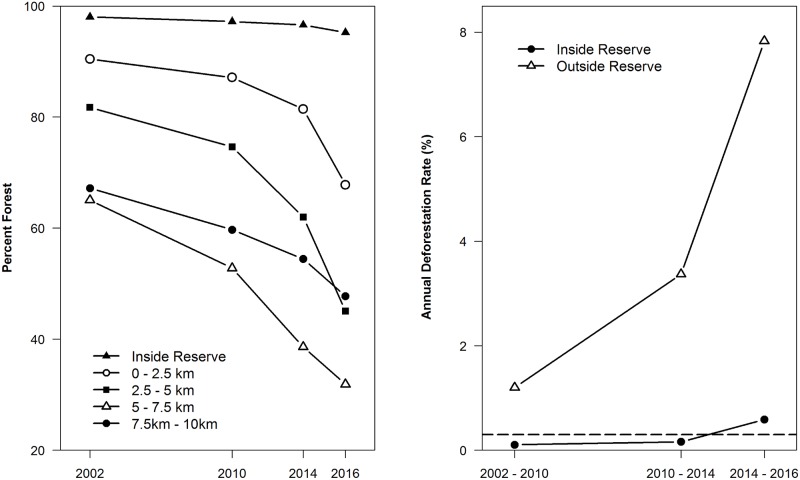
Percent of area forested inside the proposed Lenya National Park and at varying distances from the proposed park boundaries (left). Deforestation rates inside the proposed Lenya National Park and in surrounding areas within 10 km (right). The dotted line (right panel) indicates Myanmar’s nationwide deforestation rate between 2002 and 2014 [25].

Deforestation was widespread across our study area between 2002 and 2016 (Fig 2). During this period, forest area declined both inside and outside the government forest reserves comprising the proposed Lenya National Park (Table 1). Inside the forest reserve boundaries, total forest extent declined from 98.0% to 95.2%. Drivers of forest loss inside the reserve areas included some expansion of adjacent oil palm plantation, extension of rubber and other agroforestry areas from Thailand, and forest clearing associated with village areas which existed prior to 2002. Although the surrounding landscape was primarily forested in late 2001, forest area declined from 76.7% to just 48.9% of the area outside the forest reserves by May 2016. Areas within 2.5 km of the forest reserve boundaries experienced declines in total forest area from 90.4% to 67.8%, while areas at greater distances from the proposed protected area showed even more widespread forest loss (Fig 3).

**Table 1:** Percent forest inside the proposed Lenya National Park and surrounding areas within 10 km (2002–2016).

Year	Inside (%)	Outside (%)
2002	98.0	76.7
2010	97.2	69.4
2014	96.6	60.0
2016	95.2	48.9

We also observed a rapid acceleration in deforestation rates everywhere within our study landscape, including within the proposed national park boundaries (Fig 3). The annual deforestation rate within the proposed park boundaries was 0.10% from 2002–2009 and 0.16% from 2010–2013, but increased considerably to 0.59% from 2014–2016. These recent (2014–2016) deforestation rates are nearly double the national average of 0.30% for the 2002–2014 period [25] and greater than six times the global average of 0.09% between 2000 and 2015 [23]. However, deforestation rates inside the proposed protected area were far surpassed by rates of forest clearing in the surrounding landscape. These areas experienced annualized rates of forest loss that increased from 1.20% between 2002 and 2009, to 3.37% from 2010–2013 and 7.83% from 2014–2016. The most recent deforestation rates were greater than 25 times the national average from 2002–2014 and greater than 80 times the global average for 2000–2015.

#### Comparison with existing data

The estimated extent of forest loss in this study largely agrees with a March 2016 land cover analysis for Tanintharyi that focused on mapping the region’s unique forest types and areas of forest degradation [24]. Within the proposed protected areas and surrounding 10 km buffer, just 8.6% of the area identified as deforested in the current study was classified as intact forest in the previous study. This area of intact forest within the hand-digitized deforested areas in the current study is known to include intact forest areas cleared between the final imagery dates of each study (March vs. May, 2016) but may also include inaccuracies in either dataset. Other areas identified as deforested in the current study were classified as either degraded forest (49.2%), which may include early vegetation growth in young plantations, or non-forest (42.2%). Remaining areas that were not identified as deforested in the current study were classified as 79.9% intact forest, 18.7% degraded forest, and just 1.3% non-forest in the former study [24].

### Descriptions of two new species of bent-toed geckos

Myanmar presently has 18 named species of bent-toed geckos, genus *Cyrtodactylus*. Four of these geckos occur in the forests of Mon State and Tanintharyi Region, the elongate area bordering peninsular Thailand. Two of these species (*C*. *brevipalmatus*, *C*. *oldhami*,) are moderately widespread, although neither occurs throughout this entire area. Two newly discovered, morphologically distinct populations represent new species vouchered this past June, and have molecular signatures that match no other populations of Southeast Asian *Cyrtodactylus* currently available. These new species are endangered by current deforestation of the proposed Lenya National Park and Lenya National Park Extension (Table 2).

**Table 2:** New *Cyrtodactylus* species profiles.

Name	Forest Type	Distance to Reserve Boundary	Distance to Forest Clearing
*Cyrtodactylus lenya* sp. nov.	Mature Lowland Evergreen	9.1 km	7.8 km
*Cyrtodactylus payarhtanensis* sp. nov.	Mature Lowland Evergreen	9.8 km	2.2 km

#### *Cyrtodactylus payarhtanensis* Mulcahy, Myint Kyaw Thura, and Zug, sp. nov

**Fig 4:**
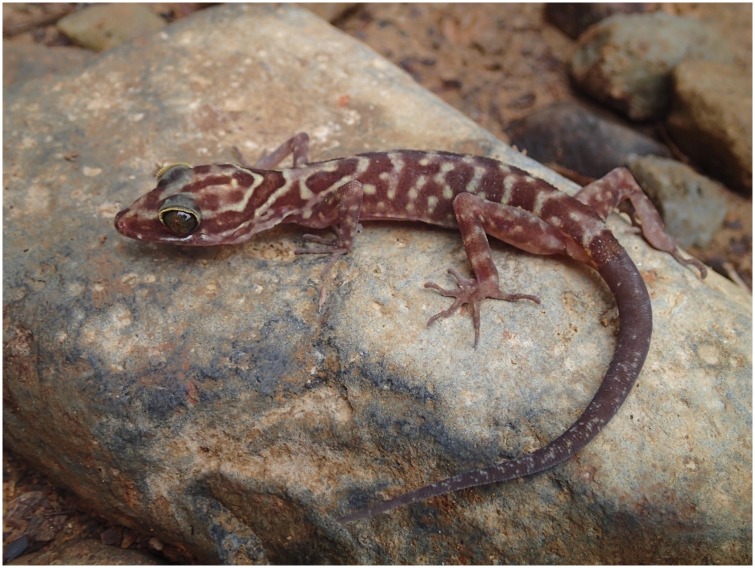
Photo of *Cyrtodactylus payarhtanensis* sp. nov., USNM 557792 paratype. (photo by Daniel G. Mulcahy).

**Tenasserim Mountain Bent-toed Gecko** (Fig 4)

urn:lsid:zoobank.org:act:454E1D2C-47C9-4A4E-B4FB-69967D511B39

*Holotype*.—USNM 587791, adult male from Myanmar, Tanintharyi Region, in the proposed Lenya National Park (11.12°N, 99.07°E), collected by Grant M. Connette and Katherine J. LaJeunesse Connette, 22 May 2016.

*Paratypes*.—CAS 260232, adult male from Myanmar, Tanintharyi Region, in the proposed Lenya National Park, collected by Myint Kyaw Thura, Thaw Zin, and Daniel G. Mulcahy on 16 May 2015; USNM 587408–409 (adult females) USNM 587410–411 (adult males), same locality and collector information as preceding paratype collected on 14 May 2015; USNM 587792, adult female, same locality data and collector information as the holotype.

*Definition*.—Midsize *Cyrtodactylus* of the *C*. *oldhami* species group, adult females 74–83 mm, males 61–80 mm SVL, possibly sexually dimorphic; 27–30% HeadL/SVL, 52–67% HeadW/HeadL, 34–46% HeadH/HeadL, 44–49% TrunkL/SVL, 14–16% ForeaL/SVL, 17–21% CrusL/SVL. Limbs slender, medium length digits of fore- and hindfeet moderate (8–10% 4FingL/SVL, 9–12% 4ToeL/SVL).

Dorsally head with granular scales, small tubercles in supratemporal area; 9–10 supralabials; 10–12 infralabials, one pair of enlarged postmentals. Dorsally trunk with 17–20 longitudinal rows of tubercles at midbody, 40–45 tubercles in paravertebral row; ventrolateral fold moderately developed and without tubercles; 26–32 ventral trunk scales at midbody smooth, overlapping and much larger than dorsal granules or tubercles. Tail with large tubercles dorsally on base, subcaudal scales distinctly enlarged, plate-like, and medially forming longitudinal row of rectangular scales. No precloacal groove or depression; distinctly enlarged row of precloacal and femoral scales but no precloacal or femoral pores; 2 cloacal spurs on each side. 5–7 proximal and 11–13 distal (16–20 total) 4FingLm; 6–8 proximal and 12–13 distal (12–13 total) 4ToeLm.

Distinctly banded dorsally and laterally, with irregularly shaped and edged dark, brown bands on neck and trunk, on a light brown background; usually six dark bands between axillary and inguinal areas. Band on posterior of neck usually present, often broken medially; band on sacrum either regular or irregular shaped; all caudal bands regular shaped, dark bands and light interspace subequal in width. Nuchal-cervical band part of postorbital stripes of light dorsal stripe above broader brown stripe; this continuous supraorbital striping and nuchal-cervical band forming U-shaped nuchal collar; nuchal band commonly notched mid-dorsally. Head indistinctly mottled dorsally, dusky brown marks on medium brown background; loreal area medium brown; supralabial and lower temporal areas medium to light brown of interspaces; limbs medium brown dorsally; venter dusky white. Preceding color description based on preserved specimens.

*Description of holotype*.—Adult male 73.4 mm SVL, 70.0 mm (regenerated) TailL; 34.5 mm TrunkL; 11.5 mm ForeaL; 8.5 mm ForefL; 5.5 mm 4FingL; 13.8 mm CrusL; 11.7 mm HindfL; 7.7 mm 4ToeL; 19.9 mmHeadL; 13.3 mm HeadW; 9.1 mm HeadH; 8.8 mm SnEye; 6.6 mm NarEye; 5.8 mm EyeEar; 6.0 mm EyeD; 6.7 mm Interorb; 2.8 mm SnW. Scalation: 9 Suplab; 7 Inflab; paired moderate large Postm; 15 DorsTub; 41 TubNum; 29 VntlSR; enlarged rectangular subcaudal scales; no precloacal or femoral pores, although continuous row of enlarged precloacal and femoral scale; 2 CloacSp; 5 4FingLmP; 10 4FingLmD; 6 4ToeLmP; 11 4ToeLmD.

*Distribution*.—This species is known only from the type locality. Southern Tenasserim Mountains within the proposed Lenya National Park, Tanintharyi Region, Myanmar.

*Etymology*.—The specific name derives from the karst landscape occupied by this species and is proposed as an adjectival noun.

*Natural history notes*.—Known only from limestone outcrops surrounded by lowland evergreen forest. Forest in the area was mostly secondary, with interspersed patches of bamboo and dipterocarp tree species.

*Comments*.—A new species, *C*. *phetchaburiensis*, recently described from two locations in adjacent Phetchaburi Province [46], Thailand likely represents a sister species, although some specimens referred to this new Thai species appear incorrectly assigned to *C*. *phetchaburiensis*. Without molecular data, we cannot define the actual relationships of this new Thai taxon and other species from southern peninsular Thailand or neighboring Myanmar.

#### *Cyrtodactylus lenya* Mulcahy, Myint Kyaw Thura, and Zug, sp. nov

**Fig 5:**
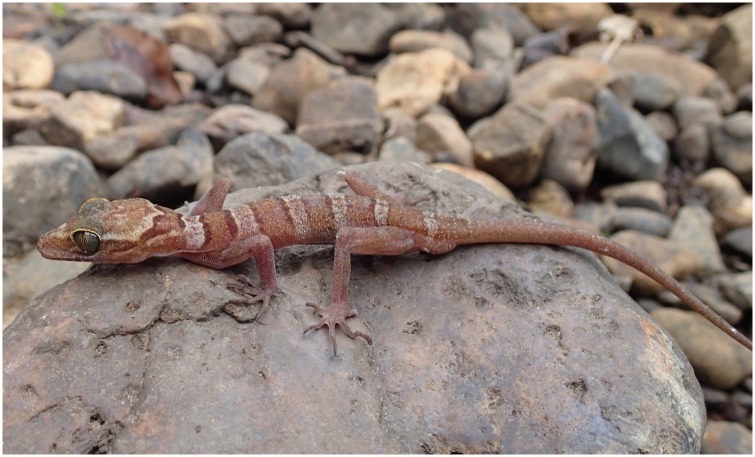
Photo of *Cyrtodactylus lenya* sp. nov., USNM 587789 paratype (photo by Daniel G. Mulcahy).

**Lenya Banded Bent-toed Gecko** (Fig 5)

urn:lsid:zoobank.org:act:95ECCE77-22D1-4692-BD99-6878D55F02B6

*Holotype*.—USNM 587788, adult female from Myanmar, Tanintharyi Region, collected in the proposed Lenya National Park Extension (11.60°N, 99.33°E) by Daniel G. Mulcahy, 15 May 2016.

*Paratypes*.—USNM 587789, adult male collected 15 May 2016 by Daniel G. Mulcahy and Grant M. Connette; CAS 260233, adult female collected 18 May 2016 by Grant M. Connette; both specimens within the vicinity of the holotype.

*Definition*.—Midsize *Cyrtodactylus* of the *C*. *oldhami* species group, adults 73–74 mm SVL, not sexually dimorphic; 27% HeadL/SVL, 61–67% HeadW/HeadL, 41–46% HeadH/HeadL, 47% TrunkL/SVL, 16% ForeaL/SVL, 16% CrusL/SVL. Limbs slender, medium length digits of fore- and hindfeet moderate (7–9% 4FingL/SVL;10–11% 4ToeL/SVL).

Dorsally head with granular scales, small tubercles in supratemporal area; 9 supralabials; 7–10 infralabials, one pair of enlarged postmentals. Dorsally trunk with 15–19 longitudinal rows of tubercles at midbody, 39–41 tubercles in paravertebral row; ventrolateral fold present but indistinct with intermittent large scales and without tubercles; 29 ventral trunk scales at midbody smooth, overlapping and 3–4X larger than dorsal granular scales. Tail with large tubercles dorsally on base, subcaudal scales distinctly enlarged, plate-like, and medially forming longitudinal row of rectangular scales. No precloacal groove or depression; distinctly enlarged row of precloacal and femoral scales but no precloacal or femoral pores; 2 cloacal spurs on each side. 5 proximal and 10–11 distal (15–16 total) 4FingLm; 6 proximal and 10–11 distal (17–18 total) 4ToeLm, basal distal lamellae on finger and toe paired.

Distinctly banded dorsally and laterally, broad, dark, two toned bands alternating with lighter interspaces; interspaces medium to light brown and half to two-thirds width of dark bands. Dark bands with narrow chocolate brown borders fore and aft of brown band (roughly anteroposterior width of interspace; five distinct dark bands, nuchal-cervical, scapular, and three trunk; less distinct sacral band followed by unicolor and equal-width brown and medium-brown bands on tail. Nuchal-cervical band part of postorbital stripes of light dorsal stripe above broader brown stripe; this continuous supraorbital striping and nuchal-cervical band forming U-shaped nuchal collar. Dorsally head indistinctly mottled, although overall appearance nearly medium brown; loreal area medium brown; supralabial and lower temporal areas medium to light brown of interspaces; limbs medium brown dorsally; venter white. Preceding color description based on preserved specimens.

*Description of holotype*.—Adult female 69.2 mm SVL, 86 mm (regenerated) TailL; 39.6 mm TrunkL; 9.7 mm ForeaL; 7.7 mm ForefL; 4.6 mm 4FingL; 12.1 mm CrusL; 10.7 mm HindfL; 7.9 mm 4ToeL; 18.5 HeadL; 11.3 mm HeadW; 6.8 mm HeadH; 4.7 mm SnEye; 6.2 mm NarEye; 4.7 mm EyeEar; 6.0 mm EyeD; 5.0 mm Interorb; 2.5 mm SnW. Scalation: 7 Suplab; 8Inflab; paired moderate large Postm; 13 DorsTub; 26 TubNum; 25 VntlSR; enlarged rectangular subcaudal scales; no precloacal or femoral pores, although continuous row of enlarged precloacal and femoral scale; 2 CloacSp; 4 4FingLmP; 12 4FingLmD; 6 4ToeLmP; 13 4ToeLmD.

*Distribution*.—The species is known only from the type locality at a single karst formation in the proposed Lenya National Park Extension in southern Tanintharyi Region, Myanmar.

*Etymology*.—The specific name refers to this species presence in the proposed Lenya National Park. The name is proposed as a noun in apposition.

*Natural history notes*.—All individuals were found on a single karst formation at elevations between 40 and 75 m. Surrounding areas were mature wet evergreen forest with the age of dominant dipterocarp trees estimated at 70–100+ years.

#### Morphological comparisons to malayan and peninsular Thailand *Cyrtodactylus*

*Cyrtodactylus lenya* and *C*. *payarhtanensis* appear to be members of the *C*. *oldhami* group of species that also includes *C*. *oldhami*, *C*. *phetchaburiensis*, C. *peguensis*, and *C*. *tigroides*. This group of mid-sized geckos (adults 50–80 mm SVL) is characterized by absence of a precloacal groove, presence of pubic patch of enlarged scales, no or few (0–8) precloacal pores, longitudinal row of enlarged precloacal and femoral scales, moderate to distinct ventrolateral trunk fold, enlarged rectangular subcaudal scales. Dorsal pattern is variable but all share a broad nuchal collar with dark center narrowly edged by white; the collar is continuous (part of) with the postorbital striping. *C*. *consobrinoides*, *C*. *lenya* and *C*. *tigroides* are the only *oldhami* group members sharing a regular banded pattern (bands dark centers edged fore and aft in white); the dark bands are much narrower than lighter interspaces in *C*. *consobrinoides*, *C*. *lenya* has 15 or more rows of dorsal tubercles and *C*. *tigroides* 13 rows. *C*. *payarhtaniensis* and some *C*. *phetchaburiensis* share dorsal bands of irregular shape (bands often diagonally transverse and not white edged); former lacks precloacal pores, latter with 4–6 precloacal pores in males and sometimes showing longitudinal dorsal stripes [46]. *C*. *payartanensis* is most similar to *C*. *variegatus* in dorsal color pattern but lacks preanal and femoral pores.

#### Molecular comparisons to other species of *Cyrtodactylus*

**Fig 6:**
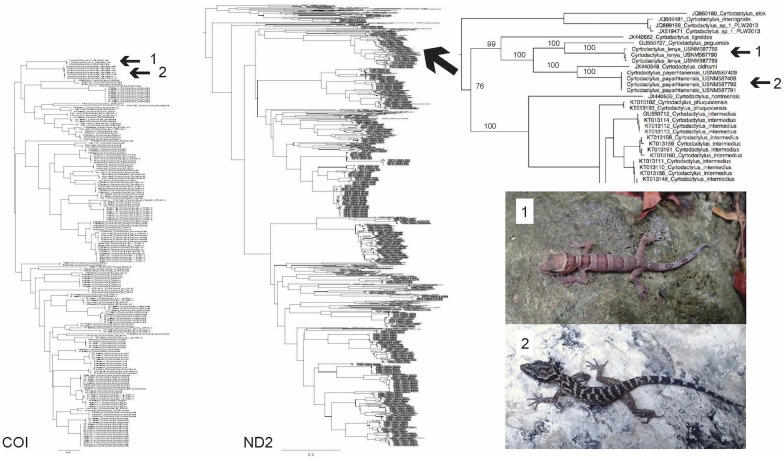
Phylogenetic placement of the two new species (1 = *Cyrtodactylus lenya* sp. nov. and 2 = *Cyrtodactylus payarhtanensis* sp. nov.) for COI (left) and ND2 (middle) mtDNA sequence data based on maximum likelihood analyses. The upper right tree shows close-up of ND2 relationships for the two new species. Middle and lower right are photos of: 1) *C*. *lenya* sp. nov. (USNM 587788; type); 2) *C*. *payarhtanensis* (USNM 587411; paratype). Both photos by Daniel G. Mulcahy.

We obtained 658 bp of the COI DNA barcode locus from all 10 new specimens of *Cyrtodactylus*. Our three *C*. *lenya* sp. nov. specimens differed from each other by 1–4 bp, the *C*. *payarhtanensis* sp. nov. differed from each other by 1–2 bp, and the two species differed by 110–113 bp (17–18% uncorrected sequence divergence). We compared sequences of our two new species with 197 other *Cyrtodactylus* COI sequences in GenBank (Fig 6). Our samples differed on an average by 20% (uncorrected) to other species in GenBank, and come out sister to each other in a clade at the base of the maximum likelihood tree, with our two new species sister to a clade containing the following species: *C*. *pulchellus*, *C*. *intermedius*, *C*. *bichnganae*, *C*. *interdigitalis*, *C*. *wayakonei*, *C*. *khasiensis*, *C*. *vilaphongi*, *C*. *otai*, *and C*. *bobrovi*, with these two clades sister to the rest of *Cyrtodactylus* with COI sequences in GenBank (Fig 6). Most inter-species relationships were poorly supported (< 50%), including the sister relationship between our two species (19%) and their relationship to the next clade (21%).

We obtained the ND2 gene region from all three of our *C*. *lenya* sp. nov. specimens ranging from 1,340–1359 bp in length (3–8 bp differences from each other) and from four of our *C*. *payarhtanensis* sp. nov. specimens (USNM 587408–09 and 587791–92) that ranged from 1,094–1,391 bp in length (0–3 bp differences from each other). The two new species range from 16–18% sequence divergence (uncorrected) from each other. We compared our sequences with 938 specimens of *Cyrtodactylus* with ND2 sequence data in GenBank. Our *C*. *lenya* sp. nov. specimens were placed sister to *C*. *peguensis* (GU550727; although we question the correct identification as *C*. *peguensis*) from Khao Luang National Park, Thailand with 100% support and were 15.3% divergent. Our *C*. *payarhtanensis* specimens were placed sister to *C*. *oldhami* (JX440548) with 100% support and were 12.5% divergent from each other. These two clades were placed sister to each other with 100% support, and were sister to *C*. *tigroides* (JX440562) with 99% support (Fig 6).

## Discussion

Myanmar’s Tanintharyi Region lies at the junction of the Indo-Burma and Sundaland biodiversity hotspots [13] and possesses a unique assemblage of both locally endemic and globally threatened wildlife species [28, 47]. The region also retains one of the largest primarily-contiguous intact forest areas in the country [25], making it critically important for the long-term conservation of wide-ranging “landscape species” such as tiger and Asian elephant [28]. Decades of armed conflict in the region previously restricted the rate of forest loss while limiting access for biological inventory and monitoring efforts. As a result, the region’s biodiversity remains poorly inventoried and the conservation status of many species is unknown.

Our recent herpetological surveys in Tanintharyi led to the description of two new species of bent-toed geckos, *Cyrtodactylus lenya* sp. nov and *Cyrtodactylus payarhtanensis* sp. nov., from isolated karst outcrops within the proposed Lenya National Park. Karst areas throughout Southeast Asia are known to harbor a wealth of biodiversity, including a number of recently-described species from a diverse range of taxa such as birds [48], rodents [49, 50], and lizards and snakes [36, 37]. Despite the high conservation value and tourism potential of karst formations, Myanmar’s karst areas are among the least protected in Southeast Asia [51]. Although quarrying is a major threat to karst ecosystems [30], fire and logging can also impact karst-affiliated species by changing local microclimate and plant communities [52] and driving away the mammal species that supply organic waste to guano-dependent communities [53]. A recent study reported that 16 species of karst-adapted reptiles, all described within the last decade, were at risk due to quarrying and oil palm encroachment in peninsular Malaysia [54]. Furthermore, previous studies have shown a general tendency for primary forest to support unique reptile and amphibian assemblages and to have a higher conservation value than plantation or secondary forest [55, 56]. These forms of habitat loss and alteration also represent a significant threat to a broader array of Southeast Asian wildlife, which respond more negatively to human land use than in other tropical regions [20]. As a result, ongoing deforestation within the proposed Lenya National Park likely poses a threat to *C*. *lenya* sp. nov. and *C*. *payarhtanensis* sp. nov., and potentially other endangered and yet-undescribed plant and animal species occupying the area’s lowland forests and karst formations.

Lowland areas in Southeast Asia have lost much of their historic forest extent and continue to experience high rates of deforestation [21, 22, 57]. Myanmar’s Tanintharyi Region is now unique within continental Southeast Asia due to the continued persistence of several large tracts of biologically-rich lowland wet evergreen forest [24]. Although extensive areas have already been lost or fragmented due to recent expansion of oil palm cultivation [24, 25, 29, 58], nearly 1/3 of Tanintharyi’s remaining lowland wet evergreen forest is contained within the boundaries of the proposed Lenya National Park and Lenya National Park Extension [24]. These areas are currently designated as government forest reserves and are considered critical for the preservation of the region’s unique wildlife species [28, 29]. Within the boundaries of these existing forest reserves, we found accelerating deforestation from 2002–2016, with rates of forest loss between 2014 and 2016 exceeding the national average for the 2002–2014 period [25]. Forest in the surrounding landscape was lost at even greater rates, as the forest frontier rapidly advanced towards the forest reserve boundaries. The annual deforestation rate within 10 km of the forest reserves reached new heights from 2014–2016 (7.85%). This exceeded peak rates of forest loss reported from the landscape surrounding Myanmar’s Chatthin Wildlife Sanctuary during a 32-year period of prolific deforestation (6.11% annually) [59]. Thus, it appears that lowland wet evergreen forest faces the imminent risk of loss and fragmentation in Myanmar’s Tanintharyi Region, an area that has served as one of the last strongholds for this ecosystem type in Southeast Asia.

### Management implications

Southeast Asia’s unique biodiversity is increasingly threatened by ongoing habitat loss, overhunting, and the unsustainable use of natural resources [43–45]. In contrast to the historically low levels of forest loss, recent years have seen rapid, widespread deforestation in Myanmar’s Tanintharyi Region [29]. The development and expansion of agroforestry plantations is a major driver of forest loss in the area [25], with oil palm cultivation particularly targeting biologically-diverse lowland forest [29]. Although numerous large-scale concessions were previously awarded for oil palm cultivation [31], some concessions are reportedly under review by the Myanmar government [60]. Furthermore, Myanmar is currently in the process of implementing new Environmental Impact Assessment (EIA) Procedures that will require review of large agroforestry plantations and their impacts on biodiversity [61]. Given the limited remaining extent of Tanintharyi’s intact lowland wet evergreen forest [24] and its critical biodiversity value [28], such review should prioritize increasing crop yields in previously cleared areas while ensuring protection of the few remaining tracts of lowland forest as well as key movement corridors between forested areas.

The two largest tracts of biologically-rich lowland evergreen forest in Myanmar’s Tanintharyi Region were proposed for formal Protected Area status over 12 and 14 years ago, respectively. These areas are no longer isolated from surrounding land use change and are currently experiencing forest clearing within the proposed park boundaries. Most forest clearing observed in the current study during field surveys was primarily associated with long-term plantings for betal nut cultivation rather than small-scale shifting cultivation. Recent reports also suggest that intense hunting pressure poses a risk to wildlife in the area [62]. Formal protection of the proposed Lenya National Park is critical to the future of Myanmar’s lowland wet evergreen forest and associated wildlife species. This will likely require decisive action and considerable investment of resources by the Myanmar government and conservation NGOs, as well as a willingness to engage with local communities which are partially governed by regional ethnic groups. In the interim period, there is a high risk of widespread forest clearing as an attempt to secure land tenure before the designation of an official protected area. Myanmar’s Tanintharyi Region remains the last stronghold of lowland evergreen rainforest in continental Southeast Asia, yet decisive action is needed to secure the future of these forests and their globally unique biodiversity.
